# Rethinking Long-Term PPI Therapy in GERD: A Narrative Review from a Microbial Ecology Perspective Beyond Acid Suppression

**DOI:** 10.3390/ph19050705

**Published:** 2026-04-30

**Authors:** Andrea Zanoni, Sonia Facchin, Valentina Mari, Luisa Bertin, Edoardo Vincenzo Savarino

**Affiliations:** 1 Unit of General Surgery, Rovereto Hospital Azienda Sanitaria Universitaria Integrata of Trento (ASUIT), 38068 Rovereto, Italy; andrea.zanoni@asuit.tn.it (A.Z.); valentina.mari@asuit.tn.it (V.M.); 2 Department of Surgery, Oncology and Gastroenterology (DISCOG), University of Padua, 35128 Padua, Italy; sonia.facchin@unipd.it (S.F.); luisa.bertin.1@phd.unipd.it (L.B.)

**Keywords:** esophageal microbiota, dysbiosis, probiotics, prebiotics, epithelial barrier, proton pump inhibitors, GERD

## Abstract

Gastroesophageal reflux disease (GERD) is a common chronic disorder of the upper gastrointestinal tract, traditionally explained by an acid-centric model in which gastric acid causes mucosal injury and symptoms. Proton pump inhibitors (PPIs) are the mainstay of therapy and effectively control symptoms in many patients. However, up to 50% of individuals remain symptomatic despite adequate acid suppression, suggesting that GERD is a multifactorial condition involving anti-reflux barrier dysfunction, impaired mucosal defense, immune activation, and alterations in the esophageal microbiota. This study is a narrative review aimed at evaluating current evidence on the interactions between acid suppression, esophageal microbial ecology, and host–microbe interactions in GERD, and at exploring the potential role of microbiota-targeted therapeutic strategies. The literature search was conducted using electronic databases (e.g., PubMed and Scopus), without formal time restrictions, prioritizing recent and clinically relevant studies. Evidence was qualitatively synthesized to provide an integrated overview. Recent studies suggest that the esophagus hosts a microbial ecosystem that may contribute to mucosal homeostasis. In GERD and Barrett’s esophagus, several studies report a shift toward Gram-negative anaerobic bacteria with potential pro-inflammatory activity. Long-term PPI therapy has been associated with increased gastric pH and changes in gastrointestinal microbiota composition, including a relative increase in taxa such as *Streptococcus* and *Veillonella*, and a reduction in short-chain fatty acid–producing bacteria. These alterations may be linked to dysbiosis and a possible increase in susceptibility to certain infections, although causality remains to be fully established. The main limitations of this review include its narrative design, the absence of systematic study selection, and the heterogeneity of the available evidence. Understanding the impact of acid suppression on microbial ecology may support the development of more integrated and personalized therapeutic strategies.

## 1. Introduction

Gastroesophageal reflux disease (GERD) represents a highly prevalent chronic disorder of the upper gastrointestinal tract and constitutes a major driver of long-term pharmacological intervention in clinical practice [1]. From a traditional pathophysiological perspective, GERD has been primarily conceptualized as the consequence of pathological acid exposure of the esophageal mucosa, resulting from an imbalance between aggressive luminal factors and esophageal defense mechanisms. This paradigm has strongly influenced therapeutic strategies over the past decades, positioning gastric acid suppression as the cornerstone of disease management. In this context, the introduction of proton pump inhibitors (PPIs) has profoundly reshaped the pathophysiological course of GERD, leading to unprecedented rates of symptom control and mucosal healing and establishing PPIs among the most widely prescribed drug classes worldwide [2,3]. The high clinical efficacy and favorable short-term safety profile of PPIs have, in turn, reinforced an acid-centric model of GERD pathogenesis and treatment, frequently translating into prolonged or lifelong therapy in a substantial proportion of patients [4,5,6].

However, accumulating evidence has progressively challenged the notion that acid exposure alone accounts for the heterogeneity of GERD phenotypes and clinical outcomes. Contemporary pathophysiological models depict GERD as a multifactorial disorder arising from the interplay between impaired esophagogastric junction competence, dysfunctional esophageal clearance mechanisms, altered mucosal barrier integrity, and dysregulated sensory processing along the gut–brain axis [7]. Within this framework, acid reflux represents only one component of a more complex network of determinants that govern symptom perception, tissue injury, and therapeutic responsiveness [8]. Notably, a substantial subset of patients continues to experience persistent symptoms despite effective acid suppression, underscoring the limitations of an exclusively acid-focused therapeutic approach and highlighting the contribution of non-acidic reflux components, mucosal vulnerability, and neuro-sensory amplification to disease expression [3,9,10,11,12,13,14,15].

Against this evolving background, increasing attention has been directed toward the broader biological consequences of sustained pharmacological acid suppression [16,17,18,19,20]. By inducing long-term hypochlorhydria, PPIs fundamentally alter the physicochemical environment of the upper gastrointestinal tract, thereby reshaping the ecological landscape of the gastric and esophageal microbial communities [21,22,23,24]. Experimental and clinical observations indicate that modifications in gastric acidity may influence microbial composition, density, and spatial distribution, potentially favoring the expansion of taxa normally restricted to the oral cavity or distal intestinal segments. These microbiota shifts are increasingly recognized as biologically relevant, given their potential to modulate local inflammatory responses, epithelial barrier function, and mucosal immune homeostasis [21,24,25]. Nevertheless, despite the growing body of literature linking acid suppression to microbial alterations throughout the gastrointestinal tract, the specific implications of long-term PPI therapy on the upper gastrointestinal microbiome in GERD remain incompletely characterized and insufficiently integrated into current disease models.

In this context, the present review aims to critically appraise the emerging evidence on the interactions between acid suppression, microbial ecology, and host–microbe crosstalk in GERD. By integrating pharmacological, microbiological, and pathophysiological perspectives, we seek to move beyond a purely acid-centric interpretation of GERD management and to provide a conceptual framework for re-evaluating the long-term use of PPIs considering their potential ecological and immunomodulatory consequences within the upper gastrointestinal tract.

This work was conducted as a narrative review. A literature search was performed using electronic databases (including PubMed and Scopus), focusing on studies addressing GERD pathophysiology, the impact of proton pump inhibitors on gastrointestinal microbiota, and microbiota-targeted therapeutic strategies. Studies were selected based on their relevance to the topic, including experimental studies, clinical trials, and microbiome analyses. No predefined systematic inclusion or exclusion criteria were applied, in line with the narrative nature of the review.

## 2. GERD: From an Acid-Centric View to a Multifactorial Model

GERD is a multifaceted disease with a shared underlying mechanism—the reflux of gastric content into the esophagus—yet markedly heterogeneous clinical manifestations. While some patients present predominantly with typical symptoms such as heartburn and regurgitation, others show other symptoms involving by extraesophageal symptoms including cough, globus sensation, dental erosions, thoracic pain, or arrhythmias [8,26,27,28]. This broad symptomatic spectrum reflects significant inter-individual variability and, in part, accounts for the divergent responses to treatment observed in clinical practice.

In recent years, several groups have begun to explore different treatment options for GERD, as many patients (up to 50%) continue to experience persistent GERD-related symptoms despite prolonged acid suppression with proton pump inhibitors (PPIs) [11,29,30,31]. Gastric acid refluxate consists of varying proportions of acid, bile, and pepsin, as well as food content and microbiota, and each component contributes to the pathogenesis of reflux [32]. From a pathophysiological perspective, gastroesophageal reflux disease is a “mechanical” disorder [33], meaning that the primary problem lies in the impaired effectiveness of the anti-reflux barrier. However, this definition should be interpreted within a broader and more integrated framework, as GERD is now widely recognized as a multifactorial condition. The anti-reflux barrier comprises multiple mechanisms that block or at least reduce the reflux of gastric contents. These mechanisms involve the lower esophageal sphincter (LES), the diaphragmatic crura, and the correct position of the esophago-gastric junction. While dysfunction of this barrier represents a key determinant of reflux, it does not fully explain the heterogeneity of clinical manifestations and therapeutic responses. Refluxate composition (including acid, bile, and pepsin), mucosal defense mechanisms, immune activation, and esophageal hypersensitivity all contribute to disease pathogenesis. Therefore, GERD cannot be considered a purely mechanical disorder, but rather a complex condition resulting from the interaction of mechanical, chemical, and neuro-immune factors [34,35]. Hence, reducing acid exposure addresses only part of the pathophysiological process and may not resolve the underlying mechanisms in all patients.

Part of this therapeutic variability is attributable to the clinical heterogeneity of the GERD phenotype spectrum. Approximately 70–80% of patients presenting with heartburn have no erosive mucosal lesions at endoscopy and are classified as non-erosive reflux disease (NERD) [36,37]. Within this population, three distinct functional subgroups can be identified: true NERD with excess esophageal acid burden; reflux hypersensitivity (RH), characterised by normal acid exposure but positive symptom-reflux association; and functional heartburn (FH), in which symptoms occur independently of reflux episodes [38,39,40]. Patients with RH and FH show substantially lower response rates to PPIs and typically benefit from neuromodulators, brain–gut behavioural interventions, and psychological therapies rather than escalated acid suppression [41,42]. Recognising this phenotypic heterogeneity is essential for interpreting PPI failure rates and for designing rational, individualized treatment strategies. According to the Lyon Consensus 2.0 [43], objective diagnosis of GERD relies on parameters such as acid exposure time (AET > 6%) and mean nocturnal baseline impedance (MNBI < 1500 Ω), which reflect pathological reflux burden and impaired mucosal integrity, respectively. These criteria are essential for distinguishing true GERD from functional esophageal disorders.

This phenotypic classification is particularly relevant when interpreting treatment response, as patients with reflux hypersensitivity or functional heartburn are less likely to benefit from acid suppression despite similar symptom profiles.

**Figure 1:**
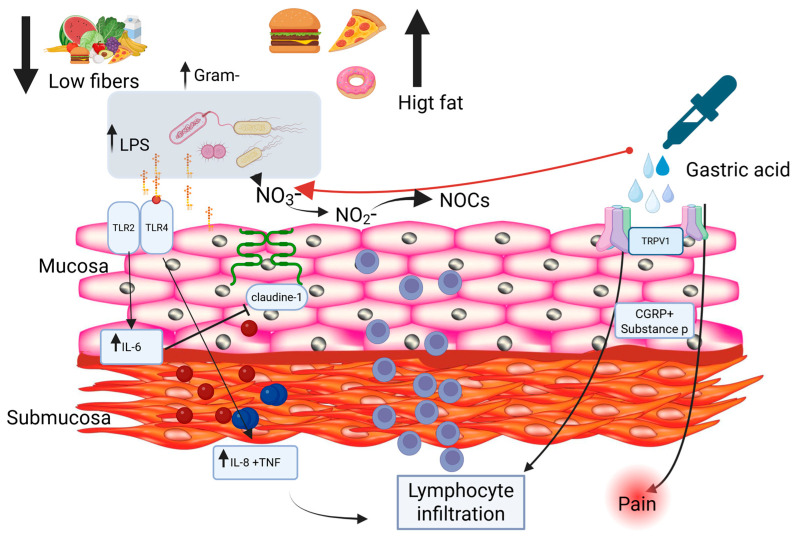
**Proposed mechanisms linking diet, microbiota alterations, and reflux-induced esophageal inflammation.** A low-fiber, high-fat diet may promote esophageal dysbiosis, characterized by an increased abundance of Gram-negative bacteria and elevated lipopolysaccharide (LPS) levels. This leads to activation of Toll-like receptors 4 and 2 (TLR4 and TLR2) in the esophageal mucosa, triggering the production of pro-inflammatory mediators, including interleukin-8 (IL-8), interleukin-6 (IL-6), and tumor necrosis factor (TNF). These processes contribute to immune cell recruitment, lymphocytic infiltration within the submucosa, and impairment of epithelial barrier integrity through downregulation of claudin-1 and dilation of intercellular spaces. In parallel, under acidic conditions, bacterial metabolism of nitrate (NO_3_−) and nitrite (NO_2_−) may promote the formation of N-nitroso compounds (NOCs), further contributing to mucosal injury. Additionally, exposure to gastric acid activates transient receptor potential vanilloid 1 (TRPV1) on sensory nerve endings, inducing the release of calcitonin gene-related peptide (CGRP) and substance P, thereby amplifying inflammation and pain signaling. The figure was created using Created in BioRender. Facchin, S. (2026) https://BioRender.com/afyi4a9, accessed on 26 April 2026.

Over the past decade, it has been shown that baseline esophageal impedance, reduced in patients with GERD, correlates with the severity of acid reflux and the integrity of the esophageal mucosa, and that patients with high baseline impedance may not benefit from PPI therapy [44,45,46,47,48,49,50,51]. Other studies have suggested that the cause of esophagitis may be cytokine-mediated. Esophageal epithelial cells may be stimulated by reflux to release cytokines, leading to recruitment of inflammatory cells that infiltrate from the submucosa upward to the epithelial surface; microscopic and histological damage may therefore appear before overt mucosal erosion [52,53]. Gastric acid activates the transient receptor potential vanilloid 1 (TRPV1) cation channel on local nerve fibers, leading to the expression of calcitonin gene–related peptide (CGRP) and substance P, which contribute to the local inflammatory response and pain signaling. Bacterial products such as lipopolysaccharides (LPS) have also been reported to bind to Toll-like receptor 4 (TLR4), stimulating IL-8 production and initiating a cascade of cytokine release, including interleukins and tumor necrosis factor (TNF). These processes result in downstream effects, including reduced sphincter relaxation and impaired gastric motility (Figure 1).

A more recent prospective study has further refined this mechanistic picture by demonstrating that TLR2, rather than TLR4, is the dominant pattern recognition receptor mediating esophageal barrier dysfunction in response to Gram-negative dysbiosis [54]. In patients with gastroesophageal reflux symptoms—whether GERD or functional esophageal disorder—esophageal microbial composition was similarly dysbiotic, characterised by a significantly elevated proportion of Gram-negative bacteria regardless of objective reflux burden, suggesting that dysbiosis may drive symptoms rather than merely reflect them. In vitro exposure of human esophageal epithelial cells to LPS upregulated TLR2 and IL-6, while downregulating claudin-1 and causing dilated intracellular spaces. Blocking TLR2 or neutralizing IL-6 both reversed these effects, establishing the LPS–TLR2–IL-6–claudin-1 axis as a key mechanistic link between Gram-negative dysbiosis and mucosal barrier dysfunction [54].

A further inflammatory pathway recently characterised involves High Mobility Group Box 1 (HMGB1), a nuclear protein released from esophageal epithelial cells under conditions of oxidative stress induced by weakly acidic bile salts. Once secreted into the extracellular space, HMGB1 activates TLR2, TLR4, and RAGE receptors on esophageal mucosal cells, triggering downstream NF-κB signalling with consequent cytokine production and increased epithelial permeability [55]. This mechanism operates independently of pH and is driven by the bile salt component of the refluxate, potentially contributing to mucosal injury and symptoms even in patients with adequate acid suppression. Notably, pre-treatment with curcumin, an antioxidant, ameliorated HMGB1 release in vitro, suggesting that antioxidant-based strategies may represent a novel therapeutic target in GERD.

Several conditions predispose to gastroesophageal reflux and may be classified as either non-modifiable or modifiable. The former include structural and physiological factors such as hiatal hernia, esophageal motility disorders, advanced age, genetic predisposition, pregnancy, and certain chronic medical conditions. Some of these factors can occasionally be addressed through surgical or pharmacological interventions, whereas others, such as chronological age and genetic predisposition, are less amenable to modification. Modifiable conditions mainly concern lifestyle and dietary habits [56,57,58,59]. The scientific literature has provided robust evidence for the association between obesity and an increased risk of reflux and its complications, as well as for the negative impact of alcohol consumption and smoking on the function of the anti-reflux barrier [60,61,62,63]. Similarly, low levels of physical activity and behaviors such as lying down immediately after meals have been identified as contributing factors [64]. Interestingly, even seemingly innocuous activities, such as prolonged television viewing, have been associated with a significant increase in risk [65].

Finally, several widely held beliefs about the causes of reflux lack adequate scientific foundation and merit clarification. Three misconceptions in particular deserve clarification. The first concerns the idea that reflux is caused by excessive gastric acidity. The primary issue is not the stomach acid itself, but its reflux into the esophagus due to a faulty anti-reflux barrier. A second misunderstanding is the belief that reflux resolves spontaneously. While an occasional episode may indeed regress without specific intervention, the chronic form tends to persist and worsen over time in the absence of appropriate lifestyle modifications and adequate treatment. Finally, there is the widespread but erroneous conviction that medications provide a definitive cure for reflux. Pharmacological treatments, although effective in relieving symptoms and reducing acid secretion, do not address the primary cause of the disorder, which may lie in anatomical defects or inappropriate behavioral habits.

## 3. Esophageal Microbial Ecology and GERD

Growing evidence indicates that microbial communities exert a relevant influence on susceptibility to several esophageal disorders, including eosinophilic esophagitis (EoE), gastroesophageal reflux disease, Barrett’s esophagus, and esophageal adenocarcinoma. The development and refinement of microbiota profiling techniques have enabled a more accurate characterization of the esophageal microbial ecosystem [66], revealing marked differences in the composition and organization of bacterial populations between physiological conditions and pathological states, thereby supporting an active involvement of the esophageal microbiota in pathogenic mechanisms [66,67,68,69,70,71,72,73,74].

**Figure 2:**
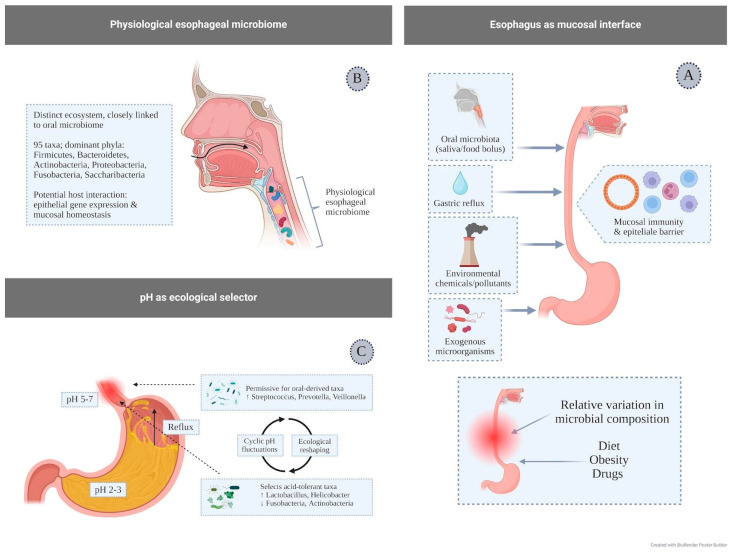
(**A**) **Factors influencing the esophageal microbiota**: oral microbiota (modulated by diet, obesity, and drugs), gastric reflux, environmental exposure, and exogenous microorganisms, promoting dysbiosis and alterations of the esophageal ecosystem with consequences for mucosal immunity. (**B**) **Physiological esophageal microbiota:** a stable microbial ecosystem, closely related to the oral microbiota, predominantly dominated by Streptococcus and involved in maintaining mucosal homeostasis. (**C**) **Esophageal reflux and pH:** fluctuations in acidity select specific microbial taxa, leading to dynamic reshaping of the esophageal microbiota. (**A**): Created in BioRender. Facchin, S. (2026) https://BioRender.com/ov9n7o9. (**B**): Created in BioRender. Facchin, S. (2026) https://BioRender.com/2tckux5. (**C**): Created in BioRender. Facchin, S. (2026) https://BioRender.com/3febga4. All accessed on 26 April 2026.

The esophagus represents a key component of mucosal immunity, acting as an initial interface between the host and multiple external stimuli; the esophageal epithelium and its immune compartment are in fact continuously exposed and responsive to dietary antigens, chemical and environmental agents, exogenous microorganisms, the oral microbiota, and gastric refluxate [75] (Figure 2A).

For a long time, the esophagus was considered a microorganism-free district; however, more recent evidence has demonstrated the existence of a specific esophageal microbial ecosystem, functionally and compositionally continuous with that of the oral cavity [66]. Under physiological conditions, this community comprises approximately 95 bacterial taxa, mainly belonging to six dominant phyla—*Firmicutes*, *Bacteroidetes*, *Actinobacteria*, *Proteobacteria*, *Fusobacteria*, and *Saccharibacteria* [76,77] (Figure 2B). Although the biological role of this microbiome has not yet been fully elucidated, experimental data suggest that the healthy esophagus harbors a relatively stable microbiota, predominantly dominated by the genus Streptococcus and characterized by the presence of other frequently detected genera, including *Actinobacillus*, *Sphingomonas*, *Neisseria*, *Haemophilus*, and *Prevotella*. These microorganisms are distributed relatively uniformly along the different segments of the esophagus, although a certain degree of interindividual specificity is observed. Overall, these findings suggest that resident microorganisms may interact with the esophageal epithelium, modulating gene expression and contributing to the maintenance of mucosal homeostasis [77].

The esophageal environment is characterized by marked physicochemical variability, which contributes to defining the ecological conditions under which the local microbiota establishes and persists. Unlike the stomach, where acidity remains constantly high, esophageal pH generally lies within a near-neutral range, although it is subject to physiological fluctuations related to food intake, swallowing, and salivary clearance [78]. In this dynamic context, pH represents a major selective determinant of the composition and function of esophageal microbial communities. Intermittent exposure to gastric contents, typical of gastroesophageal reflux, introduces episodes of transient acidification that may favor the persistence of microorganisms with higher acid tolerance, including taxa belonging to the genera *Lactobacillus* and *Helicobacter* [79]. Conversely, bacteria less adapted to low-pH conditions, such as members of the phyla *Fusobacteria* and *Actinobacteria*, tend to decrease under these circumstances [80].

Phases of more neutral or slightly alkaline pH, observed after meals or following swallowing, are instead more permissive for the colonization of oral-derived bacteria such as *Streptococcus*, *Prevotella*, and *Veillonella* [81], introduced via the alimentary bolus and salivary flow. Overall, the alternation between acidic and non-acidic conditions appears to promote a cyclical reorganization of the esophageal microbial ecosystem, with shifts in the relative dominance of different taxa according to the intensity and duration of acidifying stimuli (Figure 2C).

Scientific research has therefore increasingly focused on the microbiota of the upper gastrointestinal tract. The hypothesis has emerged that the esophageal microbiota may contribute to the pathogenesis of reflux and its complications, such as Barrett’s esophagus, which represents the precursor lesion of esophageal adenocarcinoma [82]. In the precursors of adenocarcinoma, namely GERD and Barrett’s esophagus, a transformation of the microbiota is observed, from a predominance of Gram-positive bacteria to a flora dominated by Gram-negative anaerobic or microaerophilic bacteria, including *Veillonella*, *Prevotella*, *Fusobacterium*, *Neisseria*, and *Campylobacter*. This dysbiotic condition may promote a chronic inflammatory state, facilitate metaplasia, and contribute to neoplastic progression [83,84]. The potential mechanism underlying chronic inflammation is related to bacterial products, primarily lipopolysaccharide (LPS), derived from Gram-negative bacteria. These molecules are able to activate Toll-like receptors (TLRs) on esophageal mucosal cells, triggering a cytokine- and free radical–mediated inflammatory cascade with consequent lymphocytic infiltration. Inflammation therefore does not appear to depend solely on the caustic damage induced by acid reflux, but rather emerges as a multifactorial phenomenon in which the esophageal microbiome plays a central role. In addition, potential confounding factors such as diet, obesity, medication use, and environmental exposures are often not fully controlled, making it difficult to establish independent associations between microbiota alterations and disease phenotypes.

This concept is supported by a recent study using a two-sample Mendelian randomization approach, considering the gut microbiota as the exposure variable and GERD as the outcome, which demonstrated a bidirectional interaction between dysbiosis and GERD: on the one hand, some bacterial taxa appear to exert a protective effect against gastroesophageal reflux disease (GERD), showing an inverse association with disease risk (OR < 1). These include the family *Clostridiales vadin BB60* group, genus *Lachnospiraceae UCG004*, genus *Methanobrevibacter*, and phylum *Actinobacteria*. The authors suggest that some of these taxa, particularly *Actinobacteria*, *Clostridiales Vadin*, and *Methanobrevibacter*, may contribute to protection through the production of short-chain fatty acids (SCFAs).

Conversely, other taxa appear to be associated with an increased risk of disease, including class *Mollicutes*, genus *Anaerostipes*, and phylum *Tenericutes*. Furthermore, the findings indicate that GERD itself may alter the local microenvironment, leading to changes in the composition and abundance of bacterial populations [85]. However, these findings should be interpreted with caution, as Mendelian randomization analyses rely on genetic instruments that may not fully capture the complexity of host–microbiome interactions, and residual confounding, pleiotropy, and population-specific effects may limit the strength and generalizability of causal inferences. Moreover, alterations in esophageal mucosal tight junctions may reflect a pathogenic mechanism analogous to that described for intestinal “leaky gut,” in which microbiota changes play a crucial role. In addition, animal studies have shown that LPS can induce relaxation of the lower esophageal sphincter and delay gastric emptying, both contributing factors to gastroesophageal reflux. Finally, Gram-negative bacteria can reduce dietary nitrates to nitrites, which in an acidic environment may be converted into carcinogenic N-nitroso compounds [72].

Notably, the total bacterial load does not differ significantly between healthy subjects and patients with GERD, Barrett’s esophagus, or adenocarcinoma; the critical factor therefore appears to be the relative variation in microbial composition rather than the absolute bacterial quantity. Importantly, many microbiome studies do not systematically stratify patients according to validated diagnostic criteria such as those proposed by the Lyon Consensus 2.0, which may lead to heterogeneous study populations and contribute to inconsistent findings.

Failure to distinguish between true GERD, reflux hypersensitivity, and functional heartburn may partly explain the variability in reported associations between microbiota composition and disease phenotype.

As an example, we report a study conducted by our research group in which patients with EoE were compared with non-EoE controls presenting oesophageal symptoms, mainly attributable to GERD. Multivariate analysis identified several taxa that were more represented in patients with EoE, particularly at the level of the salivary microbiota. In summary, patients with EoE showed an enrichment of taxa such as *Streptococcus cristatus*, *Prevotella oris*, *Veillonella massiliensis*, *Porphyromonas*, and *Alloprevotella*. In contrast, non-EoE controls (GERD) showed a relatively higher abundance of *Prevotella*, *Neisseria*, *Haemophilus pittmaniae*, and *Mogibacterium*. In addition, some Gram-negative genera, including *Actinobacillus*, *Porphyromonas*, and *Bergeyella*, were found to be associated with active eosinophilic inflammation [66]. Alterations of the esophageal microbiota have a multifactorial etiology, resulting from the interaction between environmental factors, pre-existing pathological conditions, and lifestyle habits. Among these, diet represents one of the most relevant determinants in shaping microbial composition. In particular, individuals adhering to a dietary pattern typical of urban settings—characterized by high intake of simple sugars, fats, and animal-derived proteins—have been reported to exhibit a higher relative abundance of Bacteroidetes and a concomitant reduction of Firmicutes in the esophageal microbiota [86]. Beyond diet, obesity worsens reflux and systemic inflammatory status, further altering the microbiota [87]. Finally, several medications can affect the microbiota, ranging from antibiotics to proton pump inhibitors. Widespread antibiotic use has been hypothesized to increase the pro-carcinogenic potential of the orodigestive microbiota. A similar argument applies to proton pump inhibitors: by reducing gastric acidity, PPI use alters the local microbial environment and, consequently, the microbiota composition [24]. It should be acknowledged that many of the available studies are observational and based on relatively small sample sizes, which may limit the robustness and generalizability of the findings. Additionally, considerable heterogeneity across microbiome studies—particularly in sampling methods, sequencing techniques, and bioinformatic pipelines—may further contribute to variability in the reported microbial profiles.

## 4. Microbiota and Proton Pump Inhibitors

The stomach physiologically produces large amounts of hydrochloric acid, resulting in a highly acidic intragastric environment with a pH well below 4. Gastric acidity serves two principal functions: digestive and protective. Hydrochloric acid and enzymes such as pepsin and lipase participate in the initial phase of digestion, while gastric juice also represents one of the most ancient defense barriers against microorganisms ingested with food.

Gastric acid secretion is an evolutionarily conserved function that first appeared in cartilaginous fish over 400 million years ago and has been maintained in amphibians, reptiles, birds, and mammals. Despite its energetic cost and its involvement in acid-related disorders such as peptic ulcer disease and gastroesophageal reflux disease, its persistence suggests a clear adaptive advantage [88].

Before the widespread use of antisecretory drugs, acquired hypo- and achlorhydria were mainly associated with chronic atrophic gastritis and malnutrition. In recent decades, however, the main causes have shifted toward the extensive use of acid-suppressive medications, particularly proton pump inhibitors (PPIs) [89]. These agents irreversibly inhibit the H^+^/K^+^-ATPase of gastric parietal cells, resulting in profound suppression of both basal and stimulated acid secretion.

PPIs are currently among the most commonly prescribed drugs worldwide and represent one of the highest-volume pharmaceutical classes in Italy. Their main indications include gastroesophageal reflux disease, peptic ulcer disease, dyspepsia, *Helicobacter pylori* eradication regimens, and prevention of ulcer-related bleeding in at-risk patients [3,19,90,91,92,93]. Nevertheless, several studies have documented widespread PPI use in the absence of appropriate indications, raising significant epidemiological, clinical, and economic concerns.

From a biological standpoint, suppression of gastric acid represents a major alteration of a key physiological and ecological barrier. Unlike most pharmacological agents, antisecretory drugs nearly abolish gastric acid secretion, impairing both digestive and protective functions of the stomach and potentially increasing susceptibility to infection. This concept is supported by a systematic review and meta-analysis showing that acid suppression is associated with an increased risk of intestinal colonization by multidrug-resistant microorganisms (OR ≈ 1.7) [94].

Acid suppression induced by PPIs alters one of the main ecological determinants of the gastrointestinal tract: the pH gradient. When intragastric pH is persistently elevated, bacteria originating from the oropharyngeal axis can more easily survive gastric transit and redistribute along the gastrointestinal tract.

Evidence from experimental next-generation sequencing NGS-based studies indicates that the most consistent increases involve *Streptococcaceae/Streptococcus* (and, less uniformly, *Veillonellaceae/Veillonella* and *Haemophilus*), while changes in other taxa appear more heterogeneous and influenced by methodological and population differences [25]. Such microbial shifts may also be influenced by underlying patient characteristics and comorbidities, making it difficult to disentangle drug-specific effects from disease-related factors.

**Figure 3:**
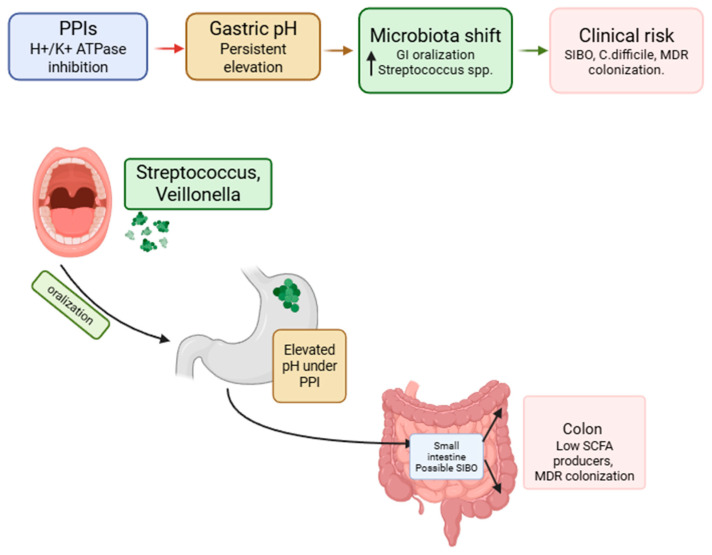
At the top, the causal flow illustrates the chain PPI → pH → bacterial shift → clinical risk; the anatomical cross-section of the GI axis visually depicts the concept of ‘oralization’—how oral-derived bacteria (Streptococcus, Veillonella) progressively colonize the stomach, small intestine, and colon as gastric pH rises. The figure was Created in BioRender. Facchin, S. (2026) https://BioRender.com/qxsavni, accessed on 26 April 2026.

During long-term treatment, persistent hypochlorhydria may act as a stable modulator of the microbiota. It promotes “oralization” of gastric and intestinal microbial communities through the selection of aerotolerant oral commensals and may be associated with signals of dysbiosis, including reduction in potentially protective taxa such as *Faecalibacterium* in some cohorts. These findings provide a biologically plausible basis for clinical associations with small intestinal bacterial overgrowth (SIBO) and enteric infections such as *Clostridioides difficile* [25,95,96] (Figure 3).

Moreover, the association between PPI-induced microbiota alterations and increased infection risk remains suggestive, as causality has not been definitively established in most available studies.

Beyond PPIs, other emerging pharmacological strategies may also have relevant—although still insufficiently characterized—effects on gastrointestinal microbial ecology. These aspects should currently be regarded as exploratory and are best interpreted as areas for future research rather than established components of GERD management.

Potassium-competitive acid blockers (P-CABs), including vonoprazan, tegoprazan, and fexuprazan, represent an important development in antisecretory pharmacology because they provide faster and more sustained acid suppression than PPIs and may offer clinical advantages in selected GERD phenotypes, particularly severe erosive esophagitis [38,97,98,99,100]. However, their ecological impact on the gastrointestinal microbiota remains largely unknown. Given their potent and prolonged acid suppression, microbiota-related effects are biologically plausible, but direct evidence is currently lacking. Therefore, the integration of P-CABs into the ecological framework discussed in this review should be considered a priority for future investigation.

Similarly, GLP-1 receptor agonists may indirectly influence GERD-related mechanisms through effects on body weight, gastric emptying, and gut microbiota composition [101]. Available evidence suggests measurable but heterogeneous microbiota changes, largely derived from preclinical models, whereas human data remains limited and associative. In GERD patients, these agents may theoretically exert both beneficial and unfavorable effects, but their clinical and ecological relevance is still uncertain. Accordingly, their role should currently be viewed as exploratory and deserving of dedicated prospective study rather than as part of the established therapeutic framework.

Taken together, these emerging therapies broaden the perspective on acid suppression and microbial ecology, but current evidence remains insufficient to support firm mechanistic or clinical conclusions.

A comparative analysis in GERD patients receiving PPI therapy suggests that both duration of exposure and current use status may be associated with distinct microbial signatures. PPI users show higher relative abundances of families such as *Streptococcaceae* and *Veillonellaceae* in feces, and differences between long-term and short-term users have been reported for several bacterial genera, suggesting that prolonged acid suppression may lead to a more structured remodeling of the gut microbiota [96].

Short-term PPI exposure (days to weeks) generally produces more modest microbiome changes. Most studies report no marked alterations in overall α- or β-diversity, although specific taxonomic shifts may occur, frequently involving increases in taxa compatible with oral microbial contribution. For example, in patients with reflux esophagitis, increases in *Lactobacillus* spp. and *Streptococcus* spp. in fecal samples have been observed within 4–8 weeks of therapy [25,102].

In this regard, a recent randomized trial comparing Omeprazole and a mucosal protective agent (PoliprotectTM) confirmed that measurable microbiota changes were induced by Omeprazole at 20 mg daily dose at 4 weeks, compared with the absence of alterations in microbiota with the mucosal protective agent. Whether these changes persist over time was beyond the scope of the study, but this trial suggests that microbiota changes occur rapidly during PPI treatment [103].

A longitudinal analysis from the Baltimore Longitudinal Study of Aging evaluated the long-term effects of PPI initiation using a target trial emulation approach [104]. Over a mean follow-up of approximately two years, PPI use was associated with increased microbial richness and compositional shifts, including enrichment of oral-derived bacteria such as *Streptococcus* species and increased abundance of potential pathobionts such as *Ruminococcus gnavus* and *Erysipelatoclostridium ramosum*. Conversely, taxa involved in short-chain fatty acid production, including *Eubacterium hallii* and *Ruthenibacterium lactatiformans*, were reduced.

After PPI discontinuation, some microbiota alterations appear to be at least partially reversible. Increased intragastric bacterial concentrations observed during therapy tend to decrease after drug withdrawal, and pediatric studies have shown recovery of α- and β-diversity following cessation, suggesting restoration of microbiome maturation processes [25,95,96,105].

These observations are largely based on associative data and should be interpreted with caution, as they do not establish a direct causal relationship between PPI use and microbiota changes. This phenomenon is consistently associated with an increase in bacteria typical of the oral microbiota throughout the gastrointestinal axis, documented in both upper GI samples (esophageal biopsies or gastric fluid) and fecal microbiota.

## 5. Prebiotics, Probiotics, Postbiotics and GERD

Several studies have increasingly suggested that probiotics, prebiotics, and postbiotics may exert beneficial effects in the management of gastroesophageal reflux disease [106]. In particular, these microbiota-targeted strategies have been investigated for their potential role in mitigating PPI-induced dysbiosis, improving symptom control, and supporting perioperative management in patients undergoing anti-reflux surgery [107].

Probiotics are internationally defined as live microorganisms—typically bacteria belonging to the genera *Lactobacillus* and *Bifidobacterium*, or yeasts such as *Saccharomyces*—which, when administered in adequate amounts, confer a health benefit on the host. To be classified as probiotics, these microorganisms must be viable, taxonomically well characterized at species and strain level, safe for human consumption, capable of surviving passage through the gastrointestinal tract, and demonstrate clinical efficacy in controlled studies [108].

Prebiotics, in contrast, are selectively fermentable substrates—such as certain oligosaccharides (e.g., FOS, GOS)—that beneficially modulate the composition and activity of the intestinal microbiota, thereby conferring health benefits to the host [109]. These are non-digestible fibers that selectively stimulate the growth of beneficial bacteria, promoting the production of short-chain fatty acids (SCFAs), such as butyrate and propionate [110]. They also regulate bile acid metabolism, contributing to the reduction of bile reflux and associated mucosal injury.

Synbiotics are products that combine probiotics and prebiotics within a single formulation, allowing the two components to act synergistically.

Finally, postbiotics comprise microbial metabolic products and inactivated cellular components of probiotic microorganisms, which can modulate the immune response, enhance intestinal barrier function, and exert antimicrobial effects, despite not containing live microorganisms [111].

The use of probiotics in treating lower gastrointestinal tract disorders is well-established in both scientific literature and clinical practice. Conversely, there is far less evidence and research regarding how probiotics affect the upper GI tract and reflux symptoms. Only a few studies have investigated the topic, as reported by a recent systematic review, where only trials involving adults without PPI treatment were included, although the authors acknowledged that probiotics may also be useful for patients taking PPIs. Specific strains that showed the greatest positive effects included *B. bifidum* YIT 10347, *B. lactis* HN019, and *L. gasseri* LG21 [106].

A randomized, double-blind, controlled clinical trial evaluated the adjuvant therapeutic effect of Bifidobacterium animalis subsp. lactis MH-02 supplementation in patients with reflux esophagitis receiving rabeprazole as background therapy. The results showed that the combined treatment with MH-02 was associated with a faster resolution of symptoms, a higher clinical response rate (50.98% vs. 30.61%; *p* = 0.044), a significant reduction in the Gastrointestinal Symptom Rating Scale GSRS score (*p* = 0.0007), and a longer mean time to symptom recurrence (*p* = 0.0013).

Furthermore, high-throughput microbiome analyses demonstrated that the combined therapy with MH-02 led to an increase in the α-diversity of the gut microbiota (*p* = 0.001) and significant changes in microbial composition, as shown by β-diversity analysis. At the genus level, there was an increased relative abundance of Bifidobacterium, Clostridium, and Blautia, while Streptococcus and Rothia showed a significant decrease (*p* < 0.05) [112].

In a multicenter, randomized, double-blind, placebo-controlled study with a crossover design, 100 patients with GERD receiving long-term proton pump inhibitor (PPI) therapy were randomized to receive either the probiotic *Lactobacillus paracasei F19* or placebo for six months. The aim of the study was to evaluate whether probiotic supplementation could prevent the onset of gastrointestinal symptoms associated with chronic PPI therapy. Compared with patients receiving PPI plus placebo, those treated with PPI plus *Lactobacillus paracasei F19* showed a lower incidence of bloating (significant over time, *p* = 0.015), a reduction in flatulence (*p* = 0.011), lower overall symptom scores, and improvements in stool form and bowel frequency. No significant differences were observed with regard to abdominal pain.

The authors concluded that supplementation with *Lactobacillus paracasei F19* may reduce or prevent the development of intestinal symptoms in patients with GERD undergoing long-term PPI therapy, potentially by counteracting PPI-associated intestinal dysbiosis [113].

A randomized controlled clinical trial currently underway at the Mayo Clinic in Scottsdale, Arizona, is investigating the impact of *Lactobacillus rhamnosus GG* (LGG) on PPI-induced alterations of the gastrointestinal microbiota in healthy subjects. The study aims to evaluate whether supplementation with this probiotic strain can modulate or mitigate the changes in microbial composition associated with proton pump inhibitor use [114].

In this context, since various probiotic strains presented positive gastroesophageal effects, also the use of postbiotics (inactivated microorganisms or their components/metabolites) gained interest. Compared to live probiotics, postbiotics offer relevant logistical benefits, such as greater stability and shelf life. 

The heat-inactivated *Lactobacillus johnsonii* No. 1088 (LJ88) is a postbiotic with remarkable potential. Its efficacy on gastroesophageal reflux-related symptoms in volunteers with occasional symptoms was well demonstrated in one recent study. 

The combination of *Lactobacillus acidophilus* LA14 (a probiotic) [115] with soy proteins fermented by *Lactobacillus bulgaricus* and a multivitamin complex (Pilorex^®^; Bromatech, Milan, Italy) seemed to be promising for patients with mild or occasional reflux symptoms. This combination proved effective in reducing typical symptoms, such as the frequency and severity of heartburn. This improvement correlated with an increase in the patients’ quality of life and a reduction in the intake of OTC [59]. (Table 1).

Among postbiotics, short-chain fatty acids (SCFAs)—specifically butyrate, acetate, and propionate—could play a significant role [110,117,118]. Butyrate appears to be the most effective. It has been proposed that butyrate’s functions could be beneficial regarding reflux disease in modulating gastrointestinal motility, in strengthening the epithelial barrier with the increase in expression of tight junction proteins and mucin, and in decreasing NF-κB activation, thus reducing pro-inflammatory cytokines. However, direct experimental evidence in GERD patients is still lacking [119].

It is widely demonstrated that the integrity of the esophageal mucosa is crucial for defense against acid. Therefore, anything that contributes to the maintenance of its integrity or its repair is decisive [120].

Butyrate and propionate effectively restored the barrier function in the epithelium of inflamed the esophagus. This effect was confirmed by an in vitro study that investigated the influence of butyrate and propionate on IL-13-induced inflammation in esophageal epithelial cultures, which reproduced the characteristics of the stratified epithelium of the esophagus [121].

Furthermore, treatment with butyrate showed therapeutic efficacy by significantly decreasing oxidative stress and inflammation, reducing pro-inflammatory cytokines such as IL-1β and TNF-α, and promoting gastric mucosal repair in a mouse model trial.

Although the evidence in the literature appears still too weak to provide definitive therapeutic indications, these preliminary data are immensely promising.

Beyond the treatment of esophageal disorders, there is also significant surgical interest in using probiotics during the pre- and post-operative periods to reduce dysbiosis and improve procedural outcomes. While this applies to all abdominal surgery, it is particularly relevant for the gastrointestinal tract.

For instance, the issue of dysbiosis in patients who are candidates for anti-reflux surgery is highly relevant for clinical management. These patients typically have a history of chronic use of PPIs. A recent study demonstrated a 60% prevalence of dysbiosis, calculated via a breath test, in a cohort of surgical candidates. These patients presented with significantly more bloating and belching compared to those without dysbiosis. Thus, dysbiosis appeared to be an independent factor associated with regurgitation and gas-bloat syndrome, conditions that can compromise surgical outcomes [122].

The methodological rigor of microbiome studies in GERD is also shaped by how the disease is objectively defined. The Lyon Consensus 2.0 [43] provides the current international framework for the modern diagnosis of GERD, establishing acid exposure time (AET > 6%) as the primary metric for conclusive GERD off therapy, defining mean nocturnal baseline impedance (MNBI < 1500 Ω) as a validated marker of impaired esophageal mucosal integrity, and formalizing the concepts of unproven versus proven GERD to guide testing strategy and treatment [123,124]. These standards are directly relevant to microbiome research in GERD: studies that fail to phenotype patients rigorously—distinguishing true acid-burden GERD from reflux hypersensitivity or functional heartburn—will enroll heterogeneous populations, which may explain conflicting findings in the esophageal microbiota literature.

These data suggest that to achieve an optimal surgical management is crucial to reduce pre-operative dysbiosis as much as possible, by combining an appropriate dietary protocol with probiotic supplementation. Furthermore, post-operative use of probiotics could extend pre-operative benefits by helping the body overcome the surgical procedure stress.

Research on the use of probiotics before and after surgery for gastroesophageal reflux disease is still developing, though studies on abdominal, colorectal and gastric surgery produced promising results, suggesting potential benefits for reflux disease as well. Several systematic reviews and meta-analyses consistently showed that the peri-operative use of probiotics and synbiotics (a combination of probiotics and prebiotics) is an effective strategy for improving post-operative outcomes in various types of abdominal and gastrointestinal surgery [125,126].

A recent systematic review and meta-analysis evaluated whether the perioperative administration of probiotics or synbiotics could reduce postoperative infections in patients undergoing elective colorectal surgery [127]. The analysis included 28 randomized controlled trials involving 2686 patients, of whom 1273 received probiotics or synbiotics and 1296 served as controls. The probiotic formulations most frequently used contained strains belonging to the genera Lactobacillus and Bifidobacterium, often administered as multistrain preparations. Overall, the meta-analysis demonstrated that perioperative probiotic/synbiotic supplementation was associated with a significant reduction in postoperative infections, with a pooled relative risk of 0.55 (95% CI 0.41–0.74; *p* < 0.001). Taken together, these findings suggest that perioperative administration of probiotics or synbiotics may nearly halve the risk of postoperative infections following elective colorectal surgery.

Probiotics/Synbiotics also improve gastrointestinal recovery by reducing the incidence of post-operative ileus and abdominal distension [128]. While evidence for some outcomes, such as nutritional status, remains mixed, the general consensus is that probiotics and synbiotics act by modulating the intestinal immune response and increasing the production of beneficial metabolites like short-chain fatty acids. There is also evidence suggesting their role in managing post-operative pain by modulating the gut microbiome and altering pain signaling [129].

Although the protocols studied demonstrated great variability and heterogeneity, and different types of formulations have not been directly compared, synbiotics appeared to confer a more pronounced benefit than probiotics alone [130].

Finally, the effect of probiotic and synbiotic supplementation in metabolic, anthropometric and nutritional outcomes in bariatric surgery was already established. Probiotics and synbiotics appeared to offer clinically relevant, though modest, improvements in various cardiometabolic parameters and nutritional indicators [131]. Despite differences related to both patient type (severe obesity) and surgical type (gastric resections in bariatric surgery, unlike anti-reflux fundoplication, where no resections are performed), these findings addressed aspects that overlap with functional esophageal surgery—specifically anti-reflux surgery—and the results could be at least partially translated to this field.

It is worth noting, however, that the quality of supplement products is also indispensable. Beyond the safety, viability, and synergy of the bacteria, the matrix in which they are embedded is of great importance. In multi-species probiotic formulations, the matrix is a fundamental element for ensuring efficacy and stability. It is not merely a vehicle. It protects microorganisms during production, storage, and gastrointestinal transit, influencing their viability, metabolic activity, and functional release in the intestine. Different types of matrices offer specific advantages: powders and capsules ensure high concentrations of live bacteria and mechanical protection; functional and fermented foods promote synergy with bioactive nutrients and higher consumer acceptance; while drinks and liquid solutions allow for rapid administration but require attention to stability and contamination. The choice of the matrix determines not only strain survival but also impacts patient compliance and clinical efficacy of the final product. The development of multi-species probiotics therefore requires a scientific and multidisciplinary approach, with particular focus on selecting strains with solid scientific evidence, experimental validation of the matrix, rigorous production standards, and design of functional synergies between strains to translate scientific data into safe and effective products for the patient. Future perspectives focus on increasingly targeted and innovative formulations capable of addressing complex conditions without compromising safety or quality [132].

From a clinical perspective, the available evidence supporting microbiota-targeted interventions in GERD remains heterogeneous and overall limited. The strength of evidence can be qualitatively stratified as low to moderate, with the most consistent data derived from small randomized controlled trials and observational studies, often characterized by variability in study design, patient selection, and probiotic strains used.

At present, routine use of probiotics, prebiotics, or postbiotics in GERD cannot be universally recommended, particularly as a primary therapeutic strategy. However, selected patient populations—such as individuals with persistent gastrointestinal symptoms during long-term PPI therapy or those with suspected dysbiosis—may potentially benefit from adjunctive microbiota modulation.

Among available approaches, probiotics have the most clinically investigated profile, although strain-specific effects and optimal treatment protocols remain to be clearly defined. Evidence for prebiotics, synbiotics, and postbiotics is emerging but still insufficient to support firm clinical recommendations.

Future research should aim to establish standardized treatment protocols, identify responder phenotypes, and generate high-quality randomized controlled trials to better define the role of microbiota-targeted therapies in GERD management. A qualitative summary of the available evidence and potential clinical applications of microbiota-targeted interventions in GERD is reported in Table 2.

## 6. Conclusions

Gastroesophageal reflux disease has long been interpreted through an acid-centric paradigm in which mucosal damage and symptom generation are primarily attributed to gastric acid refluxation, potentially in an excessive acidity setting [133]. However, accumulating evidence indicates that GERD is a multifactorial disorder involving a complex interaction between mechanical dysfunction of the anti-reflux barrier, impaired mucosal defense, immune activation, and microbial factors [8,133,134].

Recent advances in microbiome research have revealed that the esophagus hosts a structured microbial ecosystem that participates in the maintenance of mucosal homeostasis [83,135,136,137]. In GERD and its complications, including esophagitis, Barrett’s esophagus, and esophageal adenocarcinoma, this ecosystem undergoes compositional shifts, often characterized by an increased abundance of Gram-negative anaerobic bacteria, which may contribute to the activation of pro-inflammatory pathways [83]. Short-chain fatty acids, particularly butyrate, represent potential candidates of interest, although their role in GERD remains to be clearly established. The relevance of microbiota modulation extends to the surgical setting: candidates for anti-reflux surgery frequently present with pre-existing dysbiosis, mainly attributable to chronic PPI use, which may be associated with suboptimal procedural outcomes, supporting the rationale for further investigation of perioperative microbiota-targeted strategies within comprehensive surgical management approaches.

All the available evidence, including preliminary data, should prompt future research to clarify the causal relationships between microbiota alterations and GERD pathogenesis, standardize microbiome assessment methodologies, and evaluate microbial interventions in well-designed randomized clinical trials. A more integrated understanding of acid control, microbial ecology, and mucosal immunity may contribute to a more personalized and ecologically informed approach to GERD management beyond acid suppression, although current evidence remains largely associative.

Such an approach may incorporate, alongside acid suppression and microbiota-targeted interventions, the recognition of GERD phenotype heterogeneity, the use of neuromodulators and brain–gut behavioral therapies where appropriate, and careful evaluation of emerging antisecretory agents such as P-CABs and GLP-1 receptor agonists, whose ecological consequences remain to be fully characterized [58,138].

This review has several limitations that should be acknowledged. First, it is a narrative review, and therefore the selection and interpretation of the literature were not based on a predefined systematic methodology, which may introduce potential selection bias. Second, the available studies investigating microbiota in GERD and during proton pump inhibitor therapy are highly heterogeneous in design, including differences in sampling sites, sequencing techniques, and bioinformatic analyses, which limits the comparability of results across studies. In addition, most of the currently available evidence is derived from observational or cross-sectional studies, making it difficult to establish a clear causal relationship between microbiota alterations, GERD pathogenesis, and long-term acid suppression. Finally, although microbiota-targeted interventions such as probiotics, synbiotic, and postbiotics appear promising, the clinical evidence remains limited and heterogeneous, and larger well-designed randomized controlled trials are needed before definitive therapeutic recommendations can be made.

## Figures and Tables

**Table 1:** Clinical studies involving the use of probiotics: a compendium.

Author, Year	Country	Study Design	Population	Condition	Intervention Type	Strain/Product	Outcomes Assessed	Main Findings
**[112]**	China	Randomized double-blind controlled trial	Adults with reflux esophagitis receiving rabeprazole	GERD	Probiotic	*Bifidobacterium animalis* subsp. *lactis* MH-02	Symptom resolution, GSRS score, recurrence time, microbiota diversity	Higher clinical response (50.98% vs 30.61%, *p* = 0.044), lower GSRS *(p* = 0.0007), longer time to recurrence; ↑ α-diversity and beneficial taxa
**[113]**	Italy/Multicenter	Randomized double-blind placebo-controlled crossover trial	GERD patients on long-term PPI therapy	GERD	Probiotic	*Lactobacillus paracasei* F19	GI symptoms (bloating, flatulence), stool form and bowel frequency	Reduced bloating (*p* = 0.015), reduced flatulence (*p* = 0.011), improved bowel function
**[114]**	USA	Randomized controlled trial (ongoing)	Healthy subjects receiving PPI therapy	PPI-induced dysbiosis	Probiotic	*Lactobacillus rhamnosus* GG	Microbiota composition	Study evaluating prevention/modulation of PPI-induced microbiota alterations
**[116]**	Japan	Clinical study	Volunteers with occasional reflux symptoms	Occasional reflux	Postbiotic	Heat-inactivated *Lactobacillus johnsonii* No.1088	Reflux-related symptoms	Demonstrated improvement of reflux symptoms
**[115]**	Italy	Clinical study	Patients with mild or occasional reflux	Mild GERD	Probiotic-based formulation	*Lactobacillus acidophilus* LA14 + fermented soy proteins (*Lactobacillus bulgaricus*) + multivitamins (Pilorex^®^)	Frequency and severity of heartburn, quality of life	Reduced heartburn frequency/severity and reduced OTC medication use

*p* = *p*-value.

**Table 2:** Clinical applications of microbiota-targeted interventions in GERD.

Intervention	Level of Evidence	Key Findings	Clinical Recommendation
**Probiotics**	Low–Moderate	Small RCTs and observational studies suggest improvement in symptoms and possible modulation of dysbiosis, especially during PPI therapy. Effects are strain-specific.	May be considered as **adjunctive therapy** in selected patients (e.g., PPI users with persistent GI symptoms). Not recommended as first-line treatment.
**Prebiotics**	Low	Limited data; potential indirect benefits via SCFA production and microbiota modulation.	**Insufficient evidence** for routine use in GERD.
**Synbiotics**	Low	Emerging evidence suggests possible synergistic effects, but data is scarce and heterogeneous.	**No clear recommendation**: further studies needed.
**Postbiotics**	Low	Preliminary experimental and early clinical data suggest anti-inflammatory and barrier-enhancing effects (e.g., SCFAs such as butyrate).	Promising but **not yet supported for clinical use** in GERD.
**Overall strategy**	Low–Moderate	Evidence is heterogeneous, with variability in study design, populations, and interventions.	Microbiota-targeted therapies **should not be routinely recommended** but may be considered in **selected clinical contexts**.

## Data Availability

No new data were created or analyzed in this study. Data sharing is not applicable to this article.

## Abbreviations

The following abbreviations are used in this manuscript:

CGRP	Calcitonin gene-related peptide
CI	Confidence interval
DNA	Deoxyribonucleic acid
EoE	Eosinophilic esophagitis
FOS	Fructooligosaccharides
GERD	Gastroesophageal reflux disease
GI	Gastrointestinal
GOS	Galactooligosaccharides
GSRS	Gastrointestinal Symptom Rating Scale
H^+^/K^+^-ATPase	Hydrogen/potassium adenosine triphosphatase
IL-1β	Interleukin-1 beta
IL-8	Interleukin-8LES—Lower esophageal sphincter
LPS	Lipopolysaccharide
NF-κB	Nuclear factor kappa B
NGS	Next-generation sequencing
NOC	N-nitroso compounds
NO2−	Nitrite
NO3−	Nitrate
OR	Odds ratio
OTC	Over the counter
PPI	Proton pump inhibitor
SCFAs	Short-chain fatty acids
SIBO	Small intestinal bacterial overgrowth
TLR4	Toll-like receptor 4
TNF	Tumor necrosis factor
TRPV1	Transient receptor potential vanilloid 1
